# Olive Leaf Extract Added to Losartan Treatment Improved Klotho/Wnt/β-Catenin Signaling in Hypertensive Rats with Focal Segmental Glomerulosclerosis

**DOI:** 10.3390/antiox15010146

**Published:** 2026-01-22

**Authors:** Danijela Karanović, Nevena Mihailović-Stanojević, Milan Ivanov, Una-Jovana Vujačić, Jelica Grujić-Milanović, Maja Životić, Dragana Dekanski, Djurdjica Jovović, Zoran Miloradović

**Affiliations:** 1 Department for Cardiovascular Physiology, Institute for Medical Research, National Institute of Republic of Serbia, University of Belgrade, Dr Subotića 4, P.O. Box 39, 11129 Belgrade, Serbia; nevena@imi.bg.ac.rs (N.M.-S.); ivmilan@imi.bg.ac.rs (M.I.); unajovana@imi.bg.ac.rs (U.-J.V.); jeca@imi.bg.ac.rs (J.G.-M.); djurdjica@imi.bg.ac.rs (D.J.); zokim@imi.bg.ac.rs (Z.M.); 2 Institute of Pathology “Dr. Ðorđe Joannović”, Faculty of Medicine, University of Belgrade, Dr Subotića 1, 11000 Belgrade, Serbia; majajoker@gmail.com; 3 Department for Biology of Reproduction, Institute for the Application of Nuclear Energy, University of Belgrade, Banatska 31b, 11080 Belgrade, Serbia; dragana.dekanski@inep.co.rs

**Keywords:** Klotho, Wnt/β-catenin signaling, tempol, olive leaf extract, hypertensive rats, focal segmental glomerulosclerosis

## Abstract

The downregulation of Klotho in renal injury predicts the progression of chronic kidney disease (CKD). Klotho acts as an antagonist of the Wnt/β-catenin pathway, which is involved in the pathogenesis of proteinuria, glomerulosclerosis and tubulointerstitial fibrosis. We investigated whether losartan (L, angiotensin II type-1 receptor blocker) alone or combined with synthetic (tempol, T) or natural antioxidants (olive leaf extract, O) could alter Klotho/Wnt4/β-catenin signaling, thus reducing fibrosis and slowing the progression of focal segmental glomerulosclerosis (FSGS) in spontaneously hypertensive rats (SHR). The rats were divided into five groups. The control rats received a vehicle. The other groups received adriamycin (2 mg/kg, i.v., twice in a 3-week interval) for FSGS induction. Treatments with L, L+T and L+O (10, 10 + 100 and 10 + 80 mg/kg/day, respectively) were administered by gavage during six weeks. In the kidneys of model rats, Klotho and Wnt4 were downregulated, whereas β-catenin and fibronectin levels were increased compared with the control group. L+T did not alter Klotho, Wnt4 or fibronectin levels, while it further increased β-catenin. In contrast, L+O improved Klotho, and reduced β-catenin and fibronectin levels, although it increased PAI-1. The L+O combination reduced proteinuria more efficiently than L and decreased renal injury close to control levels. Although these findings indicate that combined treatment of losartan and olive leaf extract is promising in slowing the progression of the experimental FSGS, further clinical studies are needed to confirm its favorable outcomes and safety in CKD patients.

## 1. Introduction

Focal segmental glomerulosclerosis (FSGS) is a kidney disease with progressive glomerular scarring. Patients with FSGS constitute approximately 40% of adult cases and 20% of pediatric cases. FSGS is a common cause of nephrotic syndrome that includes proteinuria, hypoalbuminemia, hyperlipidemia and edema [1]. An experimental model of FSGS, adriamycin (ADR, doxorubicin hydrochloride)-induced nephropathy, is widely used in animal studies, adequately replicating pathophysiological features in humans: massive proteinuria, glomerulosclerosis, tubulointerstitial inflammation and fibrosis [2,3].

Hypertension and proteinuria are considered significant risk factors contributing to the progression of FSGS [1,4]. In particular, the downregulation of nitric oxide (NO) production is involved in the pathogenesis and progression of glomerular damage and proteinuria in various models of chronic kidney disease (CKD) [5,6,7]. The activity of NO is essential in vasodilatation, inflammation and tissue injury [8].

Klotho is a promising early biomarker of renal injury. Clinical studies have demonstrated that Klotho is markedly downregulated in CKD, where it predicts the progression and complications of kidney disease [9,10]. The administration of Klotho effectively reduces endogenous reactive oxygen species’ (ROS) production [11]. Previous studies have demonstrated the link between oxidative stress, Wnt/β-catenin activation and podocyte injury and dysfunction [10,12], thereby contributing to the pathogenesis of proteinuria and glomerulosclerosis [13]. Wnt proteins are highly conserved signaling molecules involved in early renal development and nephron formation [14]. Among them, Wnt4 plays a particularly important role in kidney disease, as it becomes reactivated under certain pathological conditions [15]. The Klotho activity also involves direct binding to various Wnt proteins, leading to inhibition of myofibroblast activation and fibrosis [9,13]. In the processes of nephron elongation and differentiation, Wnt4 modulates cell adhesion and migration, and regulates the mesenchymal-to-epithelial transition and tubulogenesis [16,17]. Upon binding to their receptors, Wnt proteins induce dephosphorylation and the stabilization of β-catenin, thereby stimulating the transcription of genes encoding plasminogen activator inhibitor-1 (PAI-1) and fibronectin. In the process of tissue remodeling, PAI-1 regulates fibrinolysis and proteolysis by inhibiting tissue and urokinase plasminogen activators (tPAs and uPAs). PAI-1 promotes inflammatory cell recruitment and extracellular matrix accumulation [13,18].

Angiotensin II contributes to hypertension, oxidative stress, intraglomerular hyperfiltration, tubulointerstitial inflammation and fibrosis in CKD [9]. Previously, we demonstrated the improvement in blood pressure and kidney function, and the reduction in glomerulosclerosis and tubulointerstitial lesions in the kidney of spontaneously hypertensive rats (SHR) with ADR-induced FSGS after blocking the angiotensin II type-1 receptor (AT1-R) with losartan; however, losartan failed to completely reduce kidney injury [19,20].

Tempol is a cell-permeable redox-cycling nitroxide that can act as superoxide dismutase mimetic (reduce O_2_^−^) or directly scavenge free radicals through conversion into hydroxylamine and oxoammonium [21]. Tempol showed antihypertensive effects [21] and it improved kidney function in various models of renal disease, such as cisplatin-induced nephrotoxicity and streptozotocin-induced diabetic nephropathy [22,23]. In addition to systemic oxidative stress attenuation, tempol lowered proteinuria and downregulated matrix metalloproteinase-1 in the kidneys of rats with ADR-induced FSGS, thereby repairing glomerular structure by normalizing the expression of structural proteins nestin and vimentin [7,20].

Plant extracts have attracted considerable interest as natural antioxidants, largely due to their high content of phenolic compounds [24,25,26]. Olive (*Olea europaea* L.) has been widely used in traditional medicine, and its pharmacological effects are primarily attributed to oleuropein and its derivative hydroxytyrosol [27]. Previous studies demonstrated the antioxidant activity of phenolics, and the antihypertensive, anti-atherosclerotic and antioxidant activity of triterpenoids isolated from *Olea europaea* L. leaves [28,29]. However, only a few studies described the protective effects of olive leaf extract in CKD. The administration of various doses of olive leaf extract (20, 40 and 80 mg/kg/day for 30 days) prevents lipid peroxidation and improves renal antioxidant enzyme activities, thereby indicating nephroprotection in gentamicin-induced nephrotoxicity [30]. Findings from our previous study have demonstrated that olive leaf extract (80 mg/kg, p.o., for 6 weeks) induces the strong prevention of glomerulosclerosis, interstitial inflammation and fibrosis in the experimental FSGS, without affecting antioxidant enzymes, pro-inflammatory and pro-fibrotic cytokines expressions [3]. Moreover, olive leaf extract prevents lipid peroxidation, recovers antioxidant capacity, diminishes protein oxidation and preserves antioxidant enzymes activities in the kidneys of SHR with ADR-induced FSGS [3], thereby indicating its beneficial effects because of direct free-radical scavenging activity.

To the best of our knowledge, studies on the effects of losartan treatment supplemented with olive leaf extract in the experimental CKD are lacking. Therefore, we hypothesized that losartan therapy in combination with synthetic or natural antioxidants could be more effective in preventing the processes related to progressive kidney injury.

With the aim of improving therapeutic outcomes and attenuating oxidative injuries, this study investigated whether treatment with losartan in combination with a synthetic antioxidant (tempol) or a natural antioxidant (olive leaf extract) could be more effective in reducing inflammation and fibrosis in the kidneys of SHR with FSGS and slowing the progression of FSGS. Here, we examined and compared the effects of these combined treatments on Klotho, the Wnt/β-catenin pathway, PAI-1, fibronectin, oxidative stress and antioxidant defense and NO metabolism, as well as kidney function and structural changes in SHR with FSGS.

## 2. Materials and Methods

### 2.1. Chemicals

All the chemicals used in this study were of the highest analytical grade and purchased from Sigma-Aldrich Chemical Co., St. Louis, MO, USA, unless otherwise specified. Losartan was purchased from DUP 153, Du Pont, Wilmington, DE, USA.

### 2.2. Plant Extract

Standardized dry olive leaf extract (Benolea^®^ (EFLA^®^943)) was purchased from Frutarom Switzerland Ltd. (Wadenswil, Switzerland). The extract was manufactured from the dried leaves of the plant species *Olea europaea* L. using an ethanol (80% *m*/*m*) extraction procedure, followed by a patented filtration procedure (EFLA^®^ HyperPure) and subsequent drying to yield a brown powder. The crude extract was standardized to 16–24% oleuropein and ≥30% polyphenols. The stability and microbiological purity were confirmed by the manufacturer. The quantitative analysis of the constituents of olive leaf extract by high-performance liquid chromatography (HPLC) has been reported previously [27,31,32], and they were shown to primarily consist of the phenolic oleuropein (17%), along with remaining compounds that include flavonoids, apigenine-7-O-glucoside, quercetin, and luteolin-7-O-glucoside (0.29%), tannins (0.52%) and caffeic acid (0.02%). According to the Certificate of Analysis, the batch used in the present study contained 17% *m*/*m* oleuropein, 40.5% *m*/*m* total polyphenols and 0.1% *m*/*m* residual solvent ethanol.

### 2.3. Animals and Experimental Protocol

Female SHR, 6 months old, with body weights of 180–200 g, were bred at the vivarium of the Institute for Medical Research, National Institute of Republic of Serbia, University of Belgrade. The rats were housed under standard conditions (temperature 23 ± 2 °C, humidity 55 ± 10%, and 12 h light/dark cycle) in plastic cages (four rats per cage) with free access to water and standard chow for laboratory rats (AGRO-FIRM doo, Požarevac, Serbia). The experimental protocol was in accordance with the national law on animal welfare (“Službeni Glasnik” No. 41/09) and approved by the Ethic Committee of the Institute for Medical Research and Veterinary Directorate Minister of Agriculture and Environmental Protection, Republic of Serbia (No. 323-07-00318/2015-05).

**Figure 1:**
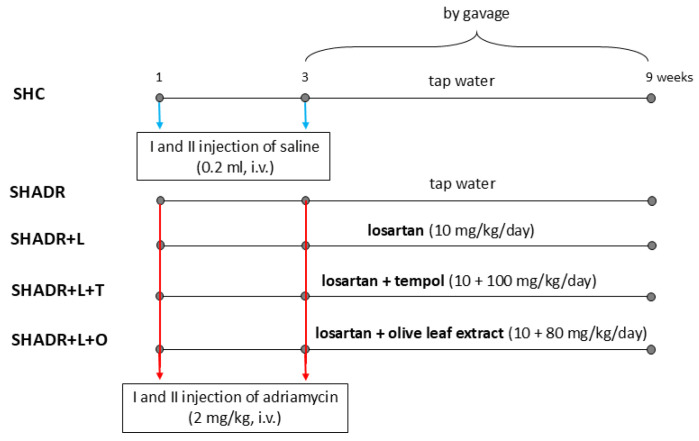
Schematic presentation of the experimental protocol. SHC, control group; SHADR, model group; L, losartan; T, tempol; and O, olive leaf extract. Blue arrows indicate saline application, and red arrows indicate adriamycin application.

The systolic blood pressure of SHR was measured by the tail cuff method (Physiograph Four, Narco Bio-system, Houston, TX, USA) and the rats were randomly divided into five groups. Adriamycin nephropathy was induced in SHR (rats were anesthetized by sodium pentobarbital, 35 mg/kg body weight, i.p.) in four groups by the intravenous injection of adriamycin in a dose of 2 mg/kg body weight, twice in a 21-day interval. Rats in the control group (SHC) received saline. After the second injection, the control and model groups (SHADR) received tap water, while the SHADR+L, SHADR+L+T and SHADR+L+O groups received L (10 mg/kg/day), L+T (10 + 100 mg/kg/day) and L+O (10 + 80 mg/kg/day) by gavage for six weeks, respectively (Figure 1).

The duration of this experimental period was determined based on our previous studies [19,20]. The doses of losartan, tempol and olive leaf extract selected for this study were in accordance with their beneficial effects and data published in our previous studies or performed by other authors [20,27,31,32]. At the end of the treatment period, the rats were placed in individual metabolic cages for 24 h urine collection. Following that, the rats were weighed and anesthetized for the measurement of hemodynamic parameters, and the collection of blood and renal tissue samples.

### 2.4. Hemodynamic Measurement

Direct measurements of hemodynamic parameters were performed in rats under anesthesia (sodium pentobarbital, 35 mg/kg body weight, i.p.) for up to 30 min. The left femoral artery was isolated and a catheter (PE–50, Clay-Adams, Parsippany, NY, USA) was placed inside to connect with the physiological data acquisition system (Cardiomax III-TCR, Thermodilution Cardiac Output, Columbus, OH, USA) for measurement of mean arterial pressure (MAP). Around the abdominal aorta, above the left renal artery bifurcation, we placed an ultrasonic flow probe (2RB) to record the aortic blood flow by Transonic T106 Small Animal Flowmeter (Transonic System Inc., Ithaca, NY, USA).

### 2.5. Sample Collection

After the hemodynamic measurement, blood samples (anticoagulant, ethylene-diamine-tetra-acetic acid disodium salt, Cleveland Inc., Cleveland, OH, USA) were taken via abdominal aorta puncture. The samples were centrifuged at 4000 rpm, 4 °C, for 20 min, and the plasma was stored at −20 °C until further analysis. Rats’ kidneys were weighed, with one half fixed in 4% formaldehyde and the other half shock-frozen in liquid nitrogen and stored at −80 °C for further analysis.

### 2.6. Biochemical Analysis

The albumin and creatinine concentrations in plasma and urine, and the protein concentration in urine were measured by an automated biochemical analyzer (COBAS INTEGRA 400 plus, Hoffmann-La Roche, Leitch Diagnostic, Penzberg, Germany). Kidney function was assessed according to the urine protein-to-creatinine ratio (U_p/cr_), protein urine excretion (P_exc_) and albumin urine excretion (Alb_exc_).

### 2.7. Measurement of Oxidative Stress Marker and Antioxidant Defense

Lipid peroxidation in plasma was assessed using the thiobarbituric acid-reactive substances (TBARS) assay [33]. The rate of TBARS production was used as a marker of oxidative stress and calculated by normalizing the TBARS concentration in plasma to aortal blood flow, expressed as nmol/min/kg.

The 2,2′-azino-bis(3-ethylbenzothiazoline-6-sulfonic acid) (ABTS•+) radical cation-based assay was used for the assessment of the antioxidant capacity in plasma and kidney homogenates [20].

The activities of the antioxidant enzymes, superoxide dismutase (SOD), catalase (CAT) and glutathione peroxidase (GP_x_), were measured in the kidney homogenates [25,34].

### 2.8. Measurement of NO Levels

The NO_x_ content, a sum of nitrate (NO_3_^−^) and nitrite (NO_2_^−^), was measured by Griess reagent method in kidney homogenates [35]. Nitrate reductase (NAD[P]H) from *Aspergillus niger* was used for conversion of nitrate to nitrite.

### 2.9. Western Blot Analysis

Kidney tissues (six rats per group) were homogenized in cold RIPA lysis buffer (50 mM Tris-HCl pH 7.5, 150 mM NaCl, 1% Triton x-100, 1% sodium deoxycholate, 0.1% sodium dodecyl sulphate, 2 mM EDTA, and 50 mM NaF, protease inhibitor cocktail (Pierce, Thermo Fisher Scientific, Waltham, MA, USA) and sodium orthovanadate), as previously described [7]. Briefly, the protein concentration was estimated by the BCA Protein Assay kit (Pierce, Thermo Scientific, Rockford, IL, USA). Equal amounts of protein (25 µg) per lane were separated by 10% SDS-PAGE and transferred onto a nitrocellulose membrane (AppliChem GmbH, Darmstadt, Germany). The membranes were blocked with 5% non-fat dry milk in Tris-buffered saline with 0.1% Tween 20 (TBS-Tween) and then incubated with the following primary antibodies: 6-nitrotryptophan (1:1000, ab243072, Abcam, Cambridge, UK), Klotho (1:1000, NBP1-76511, Novus biologicals, LCC, USA), Wnt-4 (1:2000, NBP2-20909, Novus biologicals, LCC, Centennial, CO, USA), β-catenin (1:300, AF1329, R&D Systems, Inc., Minneapolis, MN, USA), PAI-1 (1:1000, NBP1-19773, Novus biologicals, LCC, Centennial, CO, USA), fibronectin (1:1000, ab2413, Abcam, Cambridge, UK), actin (1:500, A5060, Sigma-Aldrich, MO, USA) and glyceraldehyde 3-phosphate dehydrogenase (GAPDH) (1:2500, ab9485, Abcam, Cambridge, UK). After washing with TBS-Tween, the membranes were incubated with anti-rabbit or anti-mouse IgG horseradish peroxidase-conjugated secondary antibody (A0545, A5278, Sigma-Aldrich, MO, USA). Immunoreactive bands were visualized by enhanced chemiluminescence substrate (Clarity Western ECL Blotting Substrate, Bio-Rad Laboratories, Inc., Hercules, CA, USA) and captured by the ChemiDoc Imaging system (Bio-Rad Laboratories, Inc., Hercules, CA, USA). Protein bands were quantified using Image Lab 6.0.1 software (Bio-Rad Laboratories, Inc., Hercules, CA, USA). Protein levels were normalized with respect to actin or GAPDH band density.

### 2.10. Histopathological Examination

After fixation in 4% formaldehyde, the kidney samples were rinsed in a series of ascending alcohol concentrations to dehydrate the tissue, embedded in paraffin and cut to 5 µm thick sections. Slides were stained with Periodic acid-Schiff (PAS) for histopathological examination under a light microscope (Olympus AX70, Olympus Optical Co., Ltd., Tokyo, Japan) by a pathologist blinded to the treatment group status. The severity of renal damage was assessed using a scoring system according to previous reports of renal damage [19,20]. Sclerotic changes in glomeruli were graded as 0 being normal glomeruli, 1+ being slight segmental change in small number of glomeruli, 2+ segmental and global changes in most glomeruli and 3+ general global sclerosis. Tubular dilatation with PAS+ material, atrophy of the tubular epithelium, interstitial infiltration and fibrosis were graded from 0 to 3+ according to the degree of lesions. The severity of kidney damage was determined by summing all the scores obtained for each individual parameter and presented as renal injury score. Masson’s trichrome staining of kidney slices was used to demonstrate collagen deposition in the kidney. Representative micrographs were presented at 20× magnification with 50 µm scale bar.

### 2.11. Statistical Analysis

All data are reported as mean ± standard error of mean (SEM). Statistical analysis was performed using Statistica software (version 8.0). One-way analysis of variance (ANOVA) with Fisher’s Least Significant Difference (LSD) post hoc test was used for comparison of multiple groups to evaluate statistical significance. The value of *p* < 0.05 was considered statistically significant.

## 3. Results

### 3.1. Blood Pressure, Kidney Function and Structure

**Figure 2:**
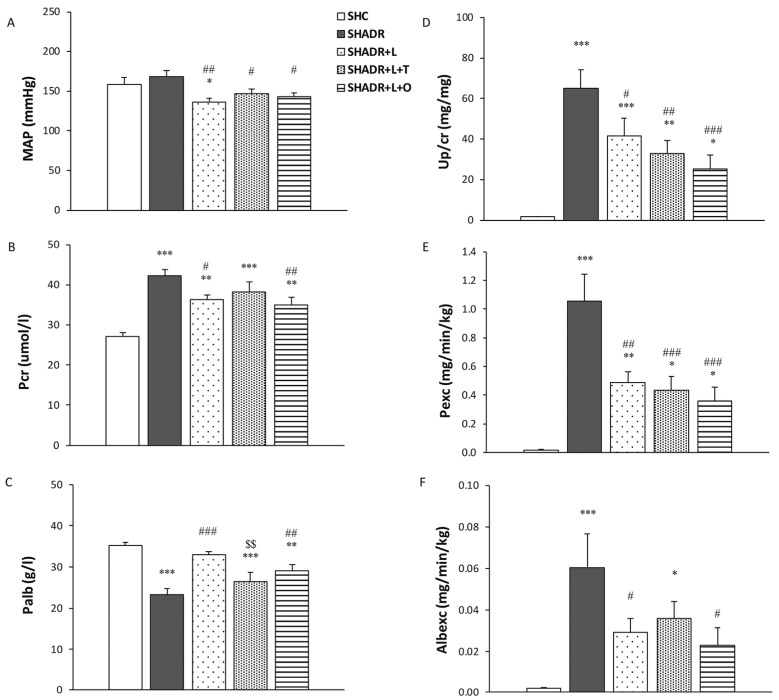
Effects of L, L+T and L+O treatments on blood pressure and kidney function in SHR with ADR-induced FSGS. (**A**) Mean arterial pressure (MAP); (**B**) plasma creatinine (P_cr_); (**C**) plasma albumin (P_alb_); (**D**) urine protein-to-creatinine ratio (U_p/cr_); (**E**) protein urine excretion (P_exc_); and (**F**) albumin urine excretion (Alb_exc_). Values represent mean ± SEM (n = 6–7). * *p* < 0.05, ** *p* < 0.01 and *** *p* < 0.001 versus SHC; # *p* < 0.05, ## *p* < 0.01 and ### *p* < 0.001 versus SHADR; and $$ *p* < 0.01 versus SHADR+L. SHC, control group; SHADR, model group; L, losartan; T, tempol; and O, olive leaf extract.

The mean arterial pressure in the model group did not differ from that in control group; however, all treatments significantly reduced blood pressure compared with the model group (Figure 2A). In addition, losartan significantly lowered blood pressure compared with control value.

The creatinine concentration in plasma was significantly increased in the SHADR group compared with the control level (Figure 2B). Only losartan and L+O treatments induced a significant reduction in creatinine level relative to the model group. The concentration of albumin in plasma was significantly decreased in the model group compared with the control group (Figure 2C). In contrast, only losartan and L+O treatments significantly increased Palb concentration compared with the level in the model group.

The Up/cr, Pexc and Albexc values were significantly increased in the model group compared with those in control group (Figure 2D–F). All treatments significantly decreased the Up/cr and Pexc in comparison to the model group, with the most pronounced reduction in protein loss accomplished by L+O treatment. The Albexc decreased significantly only after losartan and L+O treatments, reaching levels not significantly different from that of the control group.

**Figure 3:**
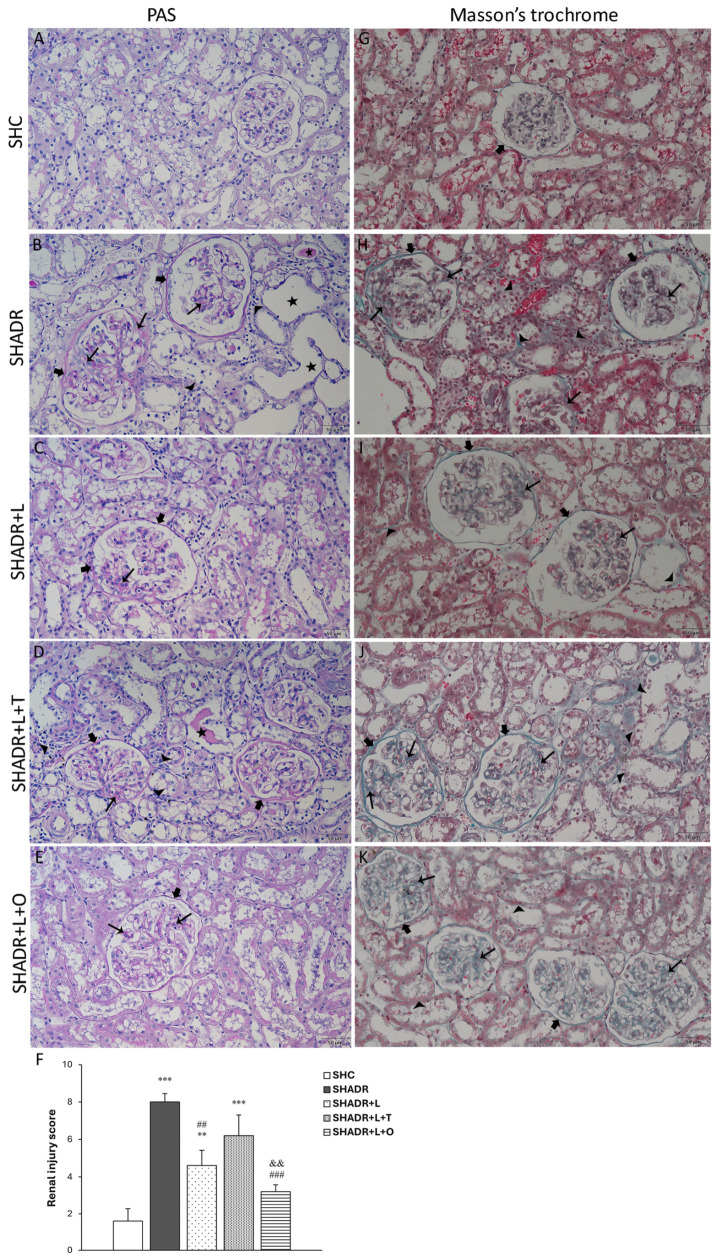
Effects of L, L+T and L+O on histology of the kidney in SHR with ADR-induced FSGS. *PAS staining* revealed the following: (**A**) SHC: normal glomeruli and tubulointerstitium. (**B**) SHADR: advanced glomerulosclerosis in lower left glomerulus with capsular adhesions of the glomerular tuft (thin arrow) and thickening Bowman’s capsule wall (bold arrow); upper right glomerulus with increased mesangial matrix (thin arrow) and thickening Bowman’s capsule (bold arrow); and tubular atrophy and dilatation (star) with PAS positive casts, periglomerular and peritubular interstitial mononuclear inflammatory infiltrate (arrowhead) and interstitial fibrosis. (**C**) SHADR+L: moderate capsular adhesion and segmental glomerular sclerosis in glomerulus (thin arrow) with thickening of Bowman’s capsule wall (bold arrow); well-preserved tubules and interstitium. (**D**) SHADR+L+T: lower glomeruli with capsular adhesion and advanced segmental glomerular sclerosis (thin arrow) and thickening of Bowman’s capsule (bold arrow); tubular dilatations with PAS positive casts (star), interstitial mononuclear inflammatory infiltrate (arrowhead) and interstitial fibrosis. (**E**) SHADR+L+O: glomerulus with mild increase in mesangial matrix (thin arrow) and less thickening of Bowman’s capsule (bold arrow); well-preserved tubules and interstitium. (**F**) Renal injury score of the PAS-stained sections. Values represent mean ± SEM (n = 5). ** *p* < 0.01 and *** *p* < 0.001 versus SHC; ## *p* < 0.01 and ### *p* < 0.001 versus SHADR; and && *p* < 0.01 versus SHADR+L+T. Masson’s trichrom staining revealed the following: (**G**) SHC: normal collagen distribution in the Bowman’s capsule of glomeruli (bold arrow); (**H**) SHADR: excessive accumulation of collagen fibers among glomerular capillaries in glomeruli (thin arrow) and in the thick Bowman’s capsule (bold arrow), and peritubular collagen deposition in the tubulointerstitium (arrowhead); (**I**) SHADR+L: moderate deposition of collagen fibers in glomeruli (thin arrow) with the thin Bowman’s capsules (bold arrow); peritubular and tubular basement membranes collagen fibers’ accumulation in interstitum (arrowhead); (**J**) SHADR+L+T: excessive deposition of collagen in glomeruli (thin arrow), in the thick Bowman’s capsule (bold arrow) and among the tubules in tubulointersititum (arrowhead); (**K**) SHADR+L+O: mild accumulation of collagen fibers (thin arrow) in the glomeruli with the thin Bowman’s capsule (bold arrow), and in tubular basement membranes in the interstitium (arrowhead). SHC, control group; SHADR, model group; L, losartan; T, tempol; and O, olive leaf extract. Scale bar, 50 µm.

The histopathological examination of PAS-stained slides revealed that normal glomeruli and tubulointerstitium were found in the kidneys of the control rats (Figure 3A). In the kidneys of SHADR rats (Figure 3B), we observed scarring of glomeruli, depicted as advanced glomerulosclerosis, along with the glomerular tuft adhesions to the thickened Bowman’s capsule. Additionally, tubular dilatation, tubular atrophy and PAS+ cast formation were found in the tubulointerstitium. The infiltration of mononuclear inflammatory cells and interstitial fibrosis were present in the early stage of ADR-induced FSGS in SHR, as we previously demonstrated [19]. Moderate glomerular changes with morphologically well-preserved tubulointerstitial compartment were observed following losartan treatment (Figure 3C). In the SHADR+L+T group we found advanced segmental glomerulosclerosis with capsular adhesion, and a similar degree of tubulointerstitial lesions, inflammation and fibrosis as in the model group (Figure 3D). In the SHADR+L+O group, a mild increase in mesangial matrix in glomeruli and well-preserved tubulointerstitium were found (Figure 3E).

The renal injury scoring revealed that glomerulosclerosis and tubulointerstitial injuries were significantly increased in ADR-treated SHR compared with control rats (Figure 3F). Losartan treatment significantly reduced renal injury in comparison to the model group; however, injury score remained significantly higher than the one observed in control rats. The L+T treatment did not produce any significant amelioration of structural changes during the early course of ADR-induced nephropathy in SHR. In contrast, L+O treatment significantly attenuated histopathological changes in the kidney compared with both the model and SHADR+L+T groups, reducing injury to a level not significantly different from those of control group.

The results from Masson’s trichrome staining revealed that fine collagen fibers were normally distributed in the Bowman’s capsules of glomeruli in the control group (Figure 3G). In contrast, the model group exhibited extensive collagen fibers’ accumulation in glomeruli, within the thicken Bowman’s capsule, and between the renal tubules (Figure 3H). A moderate deposition of collagen fibers was observed in glomeruli and tubulointerstitium following losartan treatment (Figure 3I). Rats receiving L+T treatment displayed pronounced collagen accumulation in glomeruli, within the thickening Bowman’s capsule and between the tubules (Figure 3J). In contrast, L+O-treated rats exhibited only mild collagen deposition in the glomeruli and tubulointerstitium in the kidneys (Figure 3K).

### 3.2. Oxidative Stress Marker and Antioxidant Defense

**Figure 4:**
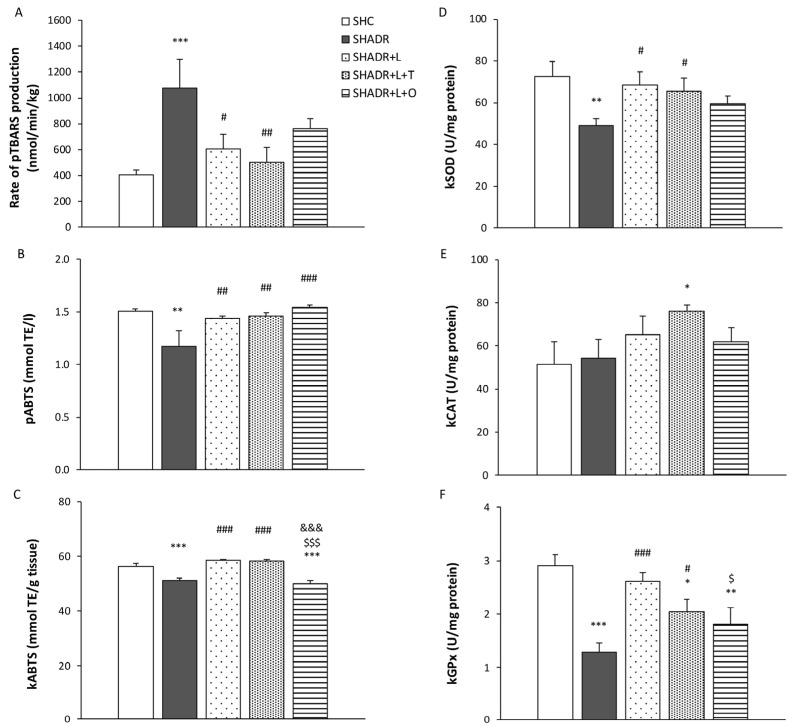
Effects of L, L+T and L+O on oxidative status, antioxidant capacity and antioxidant enzymes activities in SHR with ADR-induced FSGS. The rate of TBARS production in plasma (**A**); ABTS-antioxidant capacity of plasma (**B**); ABTS-antioxidant capacity of kidney homogenates (**C**); and the activities of the following antioxidant enzymes in kidney homogenates: superoxide dismutase (SOD) (**D**), catalase (CAT) (**E**) and glutathione peroxidase (GP_x_) (**F**). Values represent mean ± SEM (n = 6–7). * *p* < 0.05, ** *p* < 0.01, and *** *p* < 0.001 versus SHC; # *p* < 0.05, ## *p* < 0.01, and ### *p* < 0.001 versus SHADR; $ *p* < 0.05 and $$$ *p* < 0.001 versus SHADR+L; and &&& *p* < 0.001 versus SHADR+L+T. SHC, control group; SHADR, model group; L, losartan; T, tempol; O, olive leaf extract; TE, Trolox equivalent; p, plasma; and k, kidney homogenates.

Lipid peroxidation of the plasma in SHADR rats was significantly increased compared to in the control rats (Figure 4A). Treatment with losartan or the combined therapy L+T significantly reduced lipid peroxidation in comparison to the model group. Notably, plasma lipid peroxidation was reduced by 30% following L+O treatment, reaching a level that was not significantly different compared with the control group.

The level of ABTS in the plasma of rats in the model group was significantly decreased compared with control rats (Figure 4B). The antioxidant capacity of plasma was restored following all treatments. In the kidney homogenates of SHADR rats, ABTS levels were also significantly decreased; however, the renal antioxidant capacity was significantly increased after treatments with L and L+T compared with the model group (Figure 4C).

The activities of SOD and GPx in the kidneys of SHADR rats were significantly decreased in comparison to the control rats (Figure 4D,F). Treatment with losartan alone restored both enzyme activities to the control level. Combined L+T treatment significantly increased SOD and GPx activities compared with the model; however, GPx activity remained significantly lower than the control group. Following L+O treatment, SOD activity increased to a level not significantly different from the control group, whereas GPx activity remained significantly reduced. In contrast, a significant increase in CAT activity compared with the control value was observed only after L+T treatment (Figure 4E).

### 3.3. NO_3_^−^, NO_2_^−^ and NO_x_ Levels

**Figure 5:**
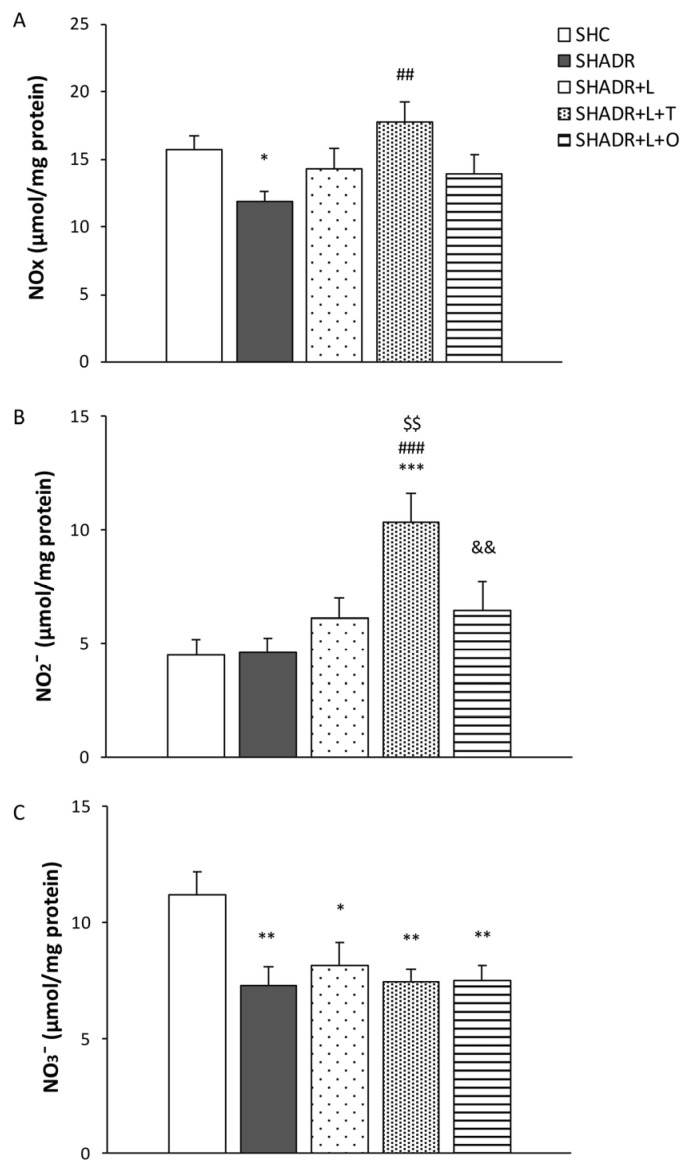
Effects of L, L+T and L+O on NO content in the kidney homogenates of SHR with ADR-induced FSGS. The NO_x_ value (**A**) represents a sum of NO_2_^−^ (**B**) and NO_3_^−^ (**C**) content. Values represent mean ± SEM (n = 6–7). * *p* < 0.05, ** *p* < 0.01 and *** *p* < 0.001 versus SHC; ## *p* < 0.01 and ### *p* < 0.001 versus SHADR; $$ *p* < 0.01 versus SHADR+L; and && *p* < 0.01 versus SHADR+L+T. SHC, control group; SHADR, model group; L, losartan; T, tempol; and O, olive leaf extract.

NO_x_ content was significantly reduced in the kidneys of SHADR rats, concomitant with a significant decrease in nitrate (NO_3_^−^) levels compared with the controls (Figure 5A,C). Treatment with losartan alone or in combination with O (L+O) restored NO_x_ levels to those observed in control rats. In contrast, T+L treatment induced a significant increase in nitrite (NO_2_^−^) levels in the kidneys, resulting in corresponding elevation of total NO_x_ content compared to the model group (Figure 5B).

### 3.4. 6-Nitrotryptophan, Klotho, Wnt-4, β-Catenin, PAI-1 and Fibronectin Protein Expressions

**Figure 6:**
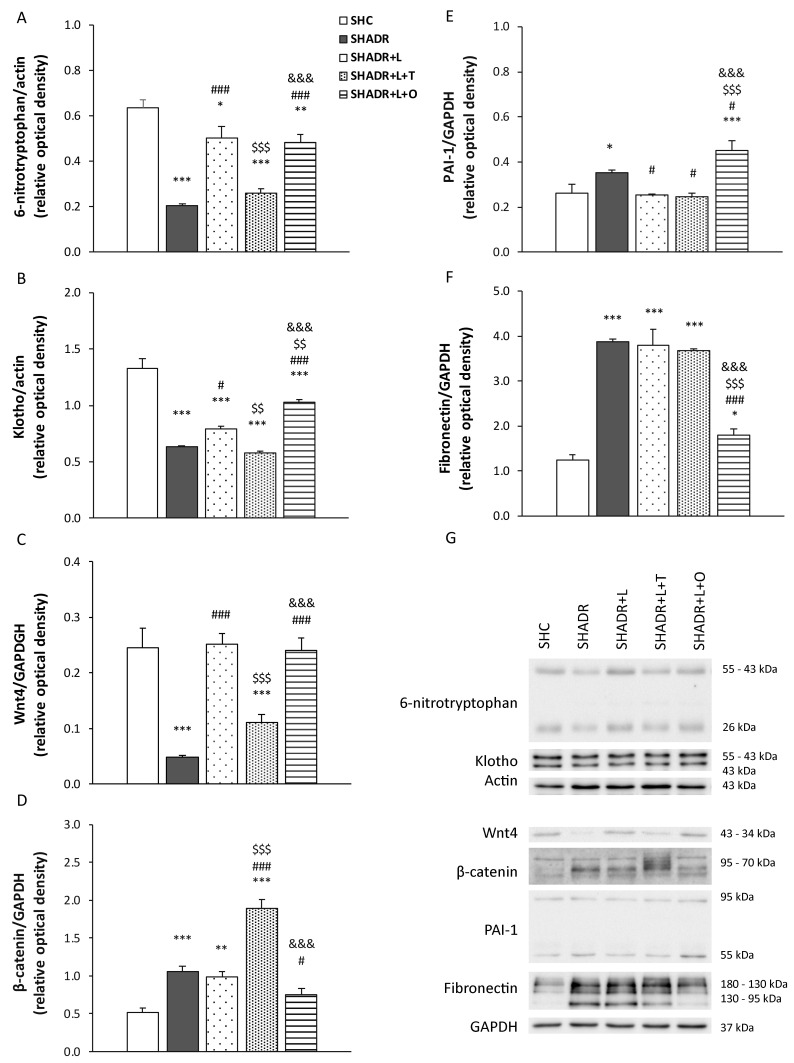
Effects of L, L+T and L+O on 6–nitrotryptophan, Klotho, Wnt4, β–catenin, PAI–1 and fibronectin protein expressions in the kidney of SHR with ADR-induced FSGS. The analysis of 6–nitrotryptophan/actin (**A**), Klotho/actin (**B**), Wnt4/GAPDH (**C**), β–catenin/GAPDH (**D**), PAI–1/GAPDH (**E**), fibronectin/GAPDH (**F**) protein expressions and representative Western blots (**G**). Values represent mean ± SEM of three to four independent experiments. * *p* < 0.05, ** *p* < 0.01 and *** *p* < 0.001 vs. SHC; # *p* < 0.05 and ### *p* < 0.001 vs. SHADR; $$ *p* < 0.01 and $$$ *p* < 0.001 vs. SHADR+L; and &&& *p* < 0.001 vs. SHADR+L+T. SHC, control group; SHADR, model group; L, losartan; T, tempol; and O, olive leaf extract.

The 6-nitrotryptophan protein expression was significantly decreased in the kidneys of SHADR rats compared with control rats (Figure 6A). Treatment with losartan alone or in combination with O (L+O) significantly increased 6-nitrotryptophan expression relative to model group; however, the levels remained significantly lower than those observed in the control group. In contrast, L+T treatment did not produce significant change in this parameter compared with the model group.

Klotho protein expression showed a trend similar to that observed for 6-nitrotryptophan. Specifically, Klotho protein expression was significantly reduced in the kidneys of SHADR rats compared with the control rats (Figure 6B). Treatment with losartan alone or in combination with O (L+O) significantly increased the Klotho level compared with the model group. L+O treatment produced a significantly stronger restorative effect than losartan alone (relative optical density of Klotho in SHADR+L+O: 1.03 ± 0.03 vs. SHADR+L: 0.80 ± 0.02, *p* < 0.01). In contrast, L+T treatment did not significantly alter Klotho protein expression compared with the model group.

The Wnt4 protein expression was significantly reduced in the kidney of model rats compared with control rats (Figure 6C). Treatment with losartan alone or in combination with O (L+O) significantly increased Wnt4 protein expression, restoring it to the control level. In contrast, L+T treatment did not induce a significant change in Wnt4 protein expression relative to the model group.

The β-catenin protein expression was significantly increased in the model group compared with the control group (Figure 6D). Treatment with losartan alone did not significantly affect β-catenin levels, whereas L+T treatment exerted a further significant increase compared with both the model and SHADR+L groups. In contrast, only L+O treatment significantly reduced β-catenin protein expression relative to the model group, restoring it to the control level.

The PAI-1 protein expression was significantly elevated in the kidneys of the model rats compared with the controls (Figure 6E). L and L+T treatments significantly reduced PAI-1 expression to the control level. Conversely, the L+O treatment induced a significant increase in PAI-1 protein expression compared with all other experimental groups.

In the kidneys of SHADR rats, fibronectin expression was significantly increased in comparison to control rats (Figure 6F). Unlike L and L+T treatments, only L+O significantly reduced fibronectin protein expression compared with the model group, even though the level remained significantly higher than in control rats.

## 4. Discussion

Our results demonstrated that losartan supplemented with natural antioxidant (olive leaf extract), in contrast to its combination with a synthetic antioxidant (tempol), induced a stronger reduction in glomerulosclerosis, tubulointerstitial inflammation and fibrosis in the kidneys of SHR with FSGS induced by ADR. This study provides insights into the major roles of Klotho and the Wnt4-independent activation of β-catenin in the process of fibrogenesis in the experimental FSGS. Moreover, these experimental findings suggest a potentially new strategy for the treatment of fibrotic kidney diseases, such as the use of AT-1 receptor blocker + olive leaf extract to slow the progression of ADR-induced FSGS in SHR.

In the kidneys of SHADR rats, we observed the development of FSGS, characterized by glomerulosclerosis, tubular injury, interstitial inflammatory cell infiltration and fibrosis, in accordance with our previous reports [7,19,20]. These structural alterations were accompanied by hypoalbuminemia, increased plasma creatinine levels, proteinuria and albuminuria, indicating impaired renal function in SHADR rats. Our findings are consistent with previous studies describing progressive renal dysfunction in this model [36,37]. Adriamycin-induced injury is closely associated with excessive reactive oxygen species (ROS) production and impaired antioxidant defense mechanisms in the kidney [38]. In the present study, adriamycin induced pronounced systemic oxidative stress, as evidenced by a significant elevation of lipid peroxidation and the reduction in the total antioxidant capacity in the plasma of the SHADR rats. In parallel, the renal antioxidant defenses of SHADR rats were significantly compromised, reflected by their decreased antioxidant capacity along with reduced SOD and GP_x_ activities. These results are consistent with previous findings [4,38,39,40]. Notably, we also detected decreased renal NO_x_ content. NO deficiency is associated with the pathogenesis of CKD and contributes to further disease progression [41,42].

The blockade of the AT-1 receptors with losartan restored NO levels, improved the antioxidant capacity and normalized antioxidant enzymes’ (SOD and GP_x_) activities in the kidney. In addition, losartan attenuated systemic oxidative stress and improved plasma antioxidant capacity, resulting in lowered blood pressure in SHADR+L rats, in agreement with earlier studies [19,20,43,44]. Furthermore, we observed an improvement in kidney function and structure after chronic losartan treatment; however, renal alterations remained more pronounced than in control rats, confirming our previous observations [19,20].

Tempol (SOD mimetic and free radical scavenger), a potent powerful redox cycling nitroxide, has been shown to promote ROS metabolism and efficiently lower blood pressure in various models of hypertension [21]. Moreover, combined treatment with tempol and losartan was previously reported to completely suppress O_2_^−^ generation in the aorta of rats with renovascular hypertension [21]. In contrast, we observed that the addition of the synthetic antioxidant tempol-to-losartan treatment failed to alleviate structural damage in the glomeruli and tubulointerstitium, showing no significant amelioration of proteinuria either. In addition, the effects of L+T treatment on antioxidant enzymes’ activities in the kidney were complex; namely, while catalase and SOD activities were increased, GP_x_ activity remained significantly reduced compared with control level. This imbalance could lead to H_2_O_2_ accumulation in the kidneys of SHADR+L+T rats. Concurrently, the NO_2_^−^ levels were elevated in the kidney. A previous study showed that NO can inactivate GP_x_ through binding to thiol groups of selenium–cysteine residues at the catalytic center [45], thereby contributing to increased H_2_O_2_ level. In addition, the reaction of SOD with H_2_O_2_ can produce damaging oxidants hydroxyl radicals (OH) [46,47], leading to NO inactivation and peroxynitrite (ONOO^−^) formation [48]. A previous study has demonstrated that nitrite and hydroxyl anions can be generated during the oxidative decomposition of peroxynitrite, prior to its isomerization to nitrate [49]. Moreover, peroxinitrites may be formed through alternative pathways, including the reaction of nitrate with H_2_O_2_ [50], as well as in reaction of SOD with NO and H_2_O_2_ under conditions of high substrate availability [48]. Consistent with these mechanisms, we also detected increased SOD activity in the kidneys of SHADR+L+T rats. In addition, the reaction of ONOO^−^ and ONOOH (peroxynitric acid) produces NO_2_^−^ and O_2_, and the yield of NO_2_^−^ increases with the increasing concentration of peroxynitrite [51]. Notably, kidney NOx content was increased in SHADR rats treated with losartan and tempol, primarily due to elevated nitrite production. Taken together, these findings suggest that an increased concentration of nitrite in the kidneys of SHADR+L+T rats could be, at least partly, due to peroxinitrite overproduction that could cause tissue injury and impaired renal function, leading to the faster progression of renal disease.

Regarding natural antioxidants, the olive leaf extract used in this study is rich in phenolic compounds, particularly oleuropein and hydroxytyrosol, which possess well-documented antioxidant, anti-inflammatory, antihypertensive and anti-atherosclerotic properties [3,27,28,29]. The extract of olive leaf is an effective radical scavenger; its major phenolic constituent, oleuropein, can act as a chelator of metal ions (e.g., Cu^2+^ and Fe^2+^) or directly neutralize free radicals via its hydroxyl groups [52], thereby limiting ROS generation. Furthermore, olive leaf extract has been shown to attenuate systemic oxidative stress through its SOD-like activity [24,30] and protects against renal oxidative stress by directly scavenging free radicals and preserving endogenous antioxidant enzymes from oxidative damage without altering their expressions [3]. Furthermore, olive leaf extract has been shown to prevent the infiltration of fibroblasts and inflammatory mononuclear cells in the kidneys of rats with ADR-induced or other types of nephropathies [30,37,53,54]. In our earlier study, we showed that the treatment with olive leaf extract strongly prevented glomerulosclerosis, interstitial inflammation and fibrosis in SHR with ADR-induced FSGS [3]. Namely, treatment with olive leaf extract normalized fibronectin, and suppressed interstitial inflammatory cells infiltration and collagen deposition, without changing cytokines expressions [3]. In the present study, combined treatment with losartan and olive leaf extract (L+O) improved SOD activity without altering GPx activity or the overall antioxidant capacity in the kidney. In addition, L+O treatment demonstrated the most pronounced trend towards the reduction in albuminuria, accompanied by the attenuation of sclerotic changes in glomeruli, tubular damage, interstitial inflammation and fibrosis in SHADR+L+O rats. Furthermore, L+O treatment prevented inactivation and normalized NO production in the kidneys. Collectively, these findings suggest that L+O treatment provides superior renoprotection compared with losartan plus tempol, likely due to the synergistic antioxidant, anti-inflammatory and antifibrotic effects of olive leaf extract in combination with the established protective actions of losartan in these experimental conditions. However, these findings are experimental and further randomized clinical outcome data and long-term safety data are needed for this combined therapy.

In the present study, the protein expression of Klotho was significantly reduced in the kidneys of SHADR rats 6 weeks after ADR administration. Consistent with our findings, a previous study has shown the downregulation of Klotho in the kidneys of mice 5 weeks after the induction of ADR nephropathy as well as in other models of CKD, e.g., unilateral ureteral obstruction (UUO), suggesting that the loss of Klotho represents a common pathological feature of kidney injury irrespective of etiology [10]. Moreover, a close association between Klotho deficiency and the robust induction of β-catenin was found in tubules in ADR nephropathy [10]. Our results are consistent with these findings. β-catenin is the principal intracellular mediator of canonical Wnt signaling and leads to the activation of a profibrogenic signaling pathway that is critically involved in the transcriptional regulation of PAI-1 and fibronectin genes [14,18]. Accordingly, concomitant with increased β-catenin expression, we also observed significant upregulation of PAI-1 and fibronectin protein expressions in the kidneys of SHADR rats. Elevated PAI-1 levels are known to promote inflammatory cell recruitment and extracellular matrix accumulation [55], consistent with the interstitial mononuclear cell infiltration and fibrosis observed in this study.

Klotho functions as an endogenous Wnt antagonist by binding multiple Wnt ligands, including Wnt1, Wnt4 and Wnt7a, thereby effectively inhibiting Wnt-mediated β-catenin activation in tubular epithelial cells in ADR nephropathy [10,13,17]. Notably, a wide range of the 19 known Wnt ligands are upregulated in kidney fibrosis [14]. Based upon downstream signaling effects, Wnt proteins have been classified into canonical, β-catenin dependent (Wnt1, 2, 3, 8a, 8b, 10a and 10b), and non-canonical, β-catenin independent (Wnt4, 5a, 5b, 6, 7a, 7b and 11) [56]. Interestingly, we observed a significant reduction in renal Wnt4 protein expression in SHADR rats, which coincided with Klotho deficiency, severe hypoalbuminemia and significant albuminuria. These results are in accordance with the previous observations from Kiewisz et al. [15], who reported a positive correlation between WNT4 gene expression and albuminemia, as well as its negative correlation with albuminuria in patients with FSGS. Despite the reduction in Wnt4 expression, we detected a strong induction of β-catenin signaling in the kidneys of SHADR rats, indicating the predominant activation of canonical rather than non-canonical Wnt signaling. Although we did not measure canonical Wnt target genes, we found increased PAI-1 and fibronectin protein expressions in the kidneys, which are consistent with the results of another study [16]. Specifically, the authors demonstrated that even though Wnt4 protein expression was reduced in the kidneys of Wnt4 knockout mice after UUO (decreased for 93% compared to control mice after UUO), the stabilization of β-catenin alone was sufficient to induce myofibroblast differentiation in interstitial pericytes and fibroblasts after UUO, providing direct evidence for the importance of the canonical Wnt/β-catenin signaling pathway in kidney fibrosis [16]. Taking together, these findings suggest that, in ADR-induced nephropathy, the upregulation of other Wnt ligands in the injured kidney may compensate for reduced Wnt4 expression and sustained β-catenin activation, thereby driving fibrotic progression in SHADR rats.

Klotho exerts well-established protective effects against myofibroblast activation and fibrosis [13]. In the present study, the chronic blockade of the AT-1 receptor with losartan significantly upregulated Klotho expression and restored Wnt4 protein levels. These findings illustrate that losartan may stimulate renal tissue repair after injury by initiating Wnt4 re-expression, a process implicated in the redifferentiation of injured tubular epithelium [57]. Namely, Wnt4 re-expression has been observed in proliferating proximal tubule epithelial cells during the repair phase following unilateral ischemia–reperfusion injury [15,57], suggesting that Wnt4 plays an important role in regulating cell cycle progression during tubular regeneration and the reconstruction of damaged tubular epithelium. In the kidneys of SHADR+L rats, we also found reduced PAI-1 protein expression without concomitant changes in β-catenin expression, indicating that PAI-1 downregulation in this context occurs independently of the β-catenin-dependent pathway. In contrast, the unchanged protein expression of fibronectin suggests that its expression may be linked to elevated β-catenin levels in the kidneys of SHADR+L rats. Recent studies have demonstrated that fibronectin contains growth factor-binding domains located near alternative splicing sites, which may modulate its functional role and potentially shift its activity towards regeneration rather than fibrotic repair [58]. Fibronectin is a dynamic component of the extracellular matrix (ECM) and plays critical roles in inflammation, cell proliferation and tissue remodeling. During the remodeling phase, the generation of the ECM is attenuated and the newly synthesized ECM undergoes continuous remodeling [58]. Taking together, these findings indicate that losartan promotes tissue regeneration to a certain degree following renal injury, thereby contributing to the structural and functional recovery of the kidney.

On the other hand, L+T treatment failed to improve Klotho and Wnt4 expression in the kidneys, while it stimulated β-catenin overexpression, which was approximately doubled compared to model rats. This pronounced β-catenin upregulation was consistent with its stabilization and was accompanied by the increased expression of fibrosis-related downstream genes, such as fibronectin. These results are consistent with previous reports demonstrating that sustained β-catenin activation drives fibrogenic signaling in renal injury [10,13,16]. Importantly, β-catenin stabilization alone has been shown to be sufficient to induce myofibroblast differentiation in interstitial pericytes and fibroblasts in renal fibrosis [16]. Similarly to our observations in the kidneys of SHADR+L rats, we also found that the downregulation of PAI-1 in the kidneys of SHADR+L+T rats occurred independently of β-catenin-dependent pathway, indicating that alternative regulatory mechanisms contribute to PAI-1 modulation under these treatment conditions.

The previous study showed the important role of Klotho in mitigating kidney injury and attenuating renal fibrosis in ADR nephropathy [59]. Klotho suppresses renal β-catenin activation and downregulates the expression of its target genes, thereby inhibiting myofibroblast activation, reducing matrix expression and ameliorating renal fibrosis [10]. In our study, L+O treatment significantly upregulated Klotho expression, which was sufficient to suppress β-catenin signaling and consequently to prevent interstitial fibronectin and collagen deposition. These molecular changes were associated with the significant amelioration of renal fibrotic lesions and a robust improvement in kidney function in SHADR+L+O rats. Additionally, L+O treatment increased renal Wnt4 protein expression. Wnt4 is a key stimulator of renal epithelial integrity, cell proliferation and mesenchymal-to-epithelial transition during nephrogenesis [17]. The re-expression of Wnt4 has been documented in proliferating proximal tubule epithelial cells during the repair phase after AKI, suggesting its essential role in tubular cell proliferation, repair and regeneration [57]. Moreover, Wnt4 re-expression following tubular epithelia injury has been shown to promote repair by antagonizing an epithelial-to-mesenchymal transition and promoting epithelial differentiation [16]. Collectively, these findings suggest that combined L+O treatment stimulates various repair mechanisms by upregulating Wnt4 protein expression in the kidneys. Our results showed that L+O treatment downregulated β-catenin, while concomitantly upregulating PAI-1 protein expression, indicating that PAI-1 regulation under these conditions occurs via mechanisms independent of β-catenin signaling. Notably, despite increased PAI-1 levels, L+O treatment significantly reduced fibrosis by preventing fibronectin and collagen deposition in the kidneys of SHADR+L+O rats. Excessive and sustained PAI-1 activity is generally associated with fibrin over-accumulation and low ECM degradation, leading to excessive collagen accumulation [55]. PAI-1 acts as a fast-acting inhibitor of tPAs, which are mainly synthesized by endothelial cells, and thereby primarily attenuates fibrinolysis. However, through the inhibition of uPA and interaction with cell surface-associated proteins (such as the uPA receptor and integrin αvβ3) and vitronectin in the ECM, PAI-1 also plays a broader role in pericellular proteolysis, tissue remodeling and cell migration [55]. Importantly, PAI-1 competes with the uPA receptor and integrin αvβ3 for binding to the same domain of vitronectin. By binding to vitronectin, PAI-1 prevents their interactions, resulting in repression of cell migration within the ECM [60]. Moreover, by binding to vitronectin, PAI-1 may prevent cardiac fibrosis and glomerulosclerosis in the kidneys. Namely, a recombinant PAI-1 variant, which only has vitronectin-binding activity but does not inhibit protease, may block integrin αvβ3 action, thereby suppress proliferation, adhesion and migration of cardiac fibroblasts [61]. Similar variants of PAI-1 protein may prevent the progression of glomerulosclerosis in diabetic nephropathy and experimental glomerulonephritis [62,63]. Namely, a noninhibitor PAI-1 variant reduces pathological ECM accumulation in large part through effectively competing with native PAI-1 for vitronectin binding sites, thereby restoring plasmin generation and increasing the plasmin-dependent degradation of matrix components [63]. Collectively, these interactions with vitronectin may be, at least partly, involved in the underling mechanisms of PAI-1 action in the kidneys of SHADR+L+O rats. On the other hand, PAI-1 is synthesized as an active molecule, which spontaneously converts into a latent form [64]. The tryptophan residues 262 and 175 are responsible for the PAI-1 protein transition from active to latent conformation [64]. Notably, nitration or the breaking of the tryptophan and histidine rings are irreversible processes, resulting in the altered conformation of proteins [65]; therefore, the modification of tryptophan residues may modulate proteins’ actions [66]. In the present study, 6-nitrotryptophan and PAI-1 expressions were enhanced in the kidneys of SHADR+L+O group compared with model group. Taken together, these findings suggest that tryptophan residues in PAI-1 may be modified after nitration, converting the PAI-1 to a latent form. If such post-translational modifications of tryptophan residues of PAI-1 have occurred under these experimental conditions, we assume that they may affect PAI-1 activity, resulting in the regression of fibrosis in SHADR+L+O rats.

In the present study, we observed renal NO_x_ content accompanied by reduced expression of 6-nitrotryptophan (a marker of protein nitration) together with pronounced tubulointerstitial inflammation in the kidneys of SHADR rats. The recruitment of inflammatory leukocytes is promoted by chemokines which are key mediators of the fibrosis-associated inflammatory response in CKD [67]. However, the previous study showed that nitration of chemokine during inflammation provides a mechanism to limit and resolve inflammation [68]. Specifically, nitrated chemokine was unable to induce trans endothelial monocyte migration in vitro and failed to effectively promote leukocyte recruitment [68]. On the other hand, studies with α-Klotho knockout mice found that the production of NO was reduced, leading to impaired endothelium vasodilation and the loss of vascular protective effects [69], underscoring the importance of NO bioavailability in maintaining renal and vascular homeostasis. In our study, only losartan and L+O treatments normalized the production of NO_x_ and restored 6-nitrotryptophan protein expression, which was associated with attenuation of tubulointerstitial inflammation in the kidneys. Taken together, these findings suggest that the restoration of NO signaling and chemokine nitration could be plausible mechanisms underlying the attenuation of inflammation, a hypothesis that warrants further investigation in future studies.

Despite our promising results, this study has several limitations which should be considered in future research. To further elucidate mechanisms underlying renal fibrosis following the treatment with losartan plus olive leaf extract, we should analyze other members of the Wnt family, specifically the Wnt1 protein. Moreover, regarding the major role of PAI-1 in renal fibrosis, the interaction of PAI-1 with vitronectin and chemokines (immunohistochemical detection in the kidneys) should be considered, as well as deeper investigation of PAI-1 activity due to the nitration of tryptophan residues. In addition, it is necessary to pay attention to the measurement of tPA or uPA activities in the kidneys. These analyses may be helpful to shade more light on the role of olive leaf extract in addition to losartan treatment in attenuating disease progression and the development of fibrosis in the experimental model of FSGS.

## 5. Conclusions

In the present study, our results showed that losartan treatment in combination with olive leaf extract (natural antioxidant) could lead to an additional decrease in fibrogenesis and improvement of renal structure (reduced glomerulosclerosis, tubulointerstitial inflammation and fibrosis) to an extent non-significantly different from control. This study provides a novel insight into the mechanism of Klotho/Wnt4/β-catenin/fibronectin signaling in the model of FSGS in response to L+O treatment. Regarding these experimental findings, adding olive leaf extract to losartan treatment may potentially slow the progression of chronic kidney disease; however, we emphasize the need for controlled clinical trials before any recommendations can be made.

## Data Availability

The original contributions presented in this study are included in the article. Further inquiries can be directed to the corresponding author.

## Abbreviations

The following abbreviations are used in this manuscript:

ABTS	2,20-azinobis-(3-ethylbenzothiazoline-6-sulfonicacid)
ADR	Adriamycin
AKI	Acute kidney injury
Albexc	Albumin urine excretion
AT-1R	Angiotensin II type-1 receptor
CKD	Chronic kidney disease
ECM	Extracellular matrix
FSGS	Focal segmental glomerulosclerosis
GAPDH	Glyceraldehyde-3-phosphate dehydrogenase
L	Losartan
MAP	Mean arterial pressure
O	Olive leaf extract
PAI-1	Plasminogen activator inhibitor-1
Palb	Plasma albumin
Pcr	Plasma creatinine
Pexc	Protein urine excretion
ROS	Reactive oxygen species
SHR	Spontaneously hypertensive rat
T	Tempol
TBARS	Thiobarbituric acid reactive substances
tPA	Tissue plasminogen activator
Up/cr	Urine protein-to-creatinine ratio
uPA	Urokinase plasminogen activator
UUO	Unilateral ureteral obstruction
